# Metal-insulator transition effect on Graphene/VO_2_ heterostructure via temperature-dependent Raman spectroscopy and resistivity measurement

**DOI:** 10.1038/s41598-024-54844-w

**Published:** 2024-02-24

**Authors:** Kittitat Lerttraikul, Wirunchana Rattanasakuldilok, Teerachote Pakornchote, Thiti Bovornratanaraks, Illias Klanurak, Thiti Taychatanapat, Ladda Srathongsian, Chaowaphat Seriwatanachai, Pongsakorn Kanjanaboos, Sojiphong Chatraphorn, Salinporn Kittiwatanakul

**Affiliations:** 1 Department of Physics, Faculty of Science, Chulalongkorn University, Bangkok, 10330 Thailand; 2 Department of Physics, Accelerator Laboratory, University of Jyväskylä, P.O. Box 35(YFL), 40014 Jyväskylä, Finland; 3 School of Materials Science and Innovation, Faculty of Science, Mahidol University, Nakhon Pathom, 73170 Thailand

**Keywords:** Materials science, Nanoscience and technology, Physics

## Abstract

High-quality VO_2_ films were fabricated on top of *c*-Al_2_O_3_ substrates using Reactive Bias Target Ion Beam Deposition (RBTIBD) and the studies of graphene/VO_2_ heterostructure were conducted. Graphene layers were placed on top of $\sim$ 50 and $\sim$ 100 nm VO_2_. The graphene layers were introduced using mechanical exfoliate and CVD graphene wet-transfer method to prevent the worsening crystallinity of VO_2_, to avoid the strain effect from lattice mismatch and to study how VO_2_ can affect the graphene layer. Slight increases in graphene/VO_2_ T_MIT_ compared to pure VO_2_ by $\sim$ 1.9 ^∘^C and $\sim$ 3.8 ^∘^C for CVD graphene on 100 and 50 nm VO_2_, respectively, were observed in temperature-dependent resistivity measurements. As the strain effect from lattice mismatch was minimized in our samples, the increase in T_MIT_ may originate from a large difference in the thermal conductivity between graphene and VO_2_. Temperature-dependent Raman spectroscopy measurements were also performed on all samples, and the G-peak splitting into two peaks, G^+^ and G^-^, were observed on graphene/VO_2_ (100 nm) samples. The G-peak splitting is a reversible process and may originates from in-plane asymmetric tensile strain applied under the graphene layer due to the VO_2_ phase transition mechanism. The 2D-peak measurements also show large blue-shifts around 13 cm^-1^ at room temperature and slightly red-shifts trend as temperature increases for 100 nm VO_2_ samples. Other electronic interactions between graphene and VO_2_ are expected as evidenced by 2D-peak characteristic observed in Raman measurements. These findings may provide a better understanding of graphene/VO_2_ and introduce some new applications that utilize the controllable structural properties of graphene via the VO_2_ phase transition.

## Introduction

Vanadium dioxide (VO_2_) has attracted considerable attention from many researchers due to its Metal-Insulator Transition (MIT) effect at the transition temperature (T_MIT_) about 68 ^∘^C under atmospheric pressure^1,2^. As temperature rises to the T_MIT_, VO_2_ features a significant change in its electrical conductivity from non-conductive material at low temperature to conductive material at high temperature by 3 orders of magnitude, along with a considerable change in its optical transmittance and reflectance^3,4^. The crystal structure of VO_2_ also changes from monoclinic (M) phase to rutile (R) phase as the rising temperature reaches the structural phase transition temperature (T_SPT_) which is around T_MIT_ in the pristine VO_2_ condition. The contrast between VO_2_ properties in these two main phases leads to various applications based on VO_2_ material, such as smart windows^5^ and electrical switch^6,7^. Various techniques were also studied in order to modify the properties related to the phase transition of VO_2_ such as refining the fabrication process^8,9^, substrate clamping^10,11^ and elemental doping^11,12^ which expand the area of its applications further. Several studies utilize the benefits of graphene, a flexible 2D material with exceptional properties, such as high light transmittance^13^ and high electron mobility^14^, to improve and introduce new applications for VO_2_/graphene heterostructure. Several studies report that graphene can improve VO_2_ characteristic by providing higher light transmission and lower phase transition temperature^15,16^. For the application, one of the interesting topics is the flexible thermochromic windows based on VO_2_/graphene proposed by Kim et al.^17^. Their result indicates that graphene is a key element that allows VO_2_ to adhere to the flexible substrate, poly(ethyleneterephthalate) (PET) film, thus expanding its smart windows application. Graphene layer in VO_2_/graphene can also be utilized in other applications, such as changing volatile to non-volatile VO_2_ transition^18^ and switchable terahertz absorber^19^. There are several previous studies about the effect between the graphene and VO_2_ layers, such as the observation of thermochromism and decreases in T_MIT_ induced by electron transfer in VO_2_–graphene–Ge heterostructure^16^ and the improvement of phase transition characteristics and optical response of VO_2_ with graphene-supported layers^15^. However, most works were studied by fabricating VO_2_ layer on top of graphene, which may lead to some limitations. Firstly, the growth process of VO_2_ requires very high temperature, which can decrease the graphene layer quality, resulting in the worsening and occurrence of poly-crystalline VO_2_^17^. Secondly, fabricating VO_2_ on top of graphene could induce a compressive strain on the VO_2_ originated from the lattice mismatch between graphene and VO_2_. The induced strain may affect the VO_2_ phase transition related properties such as the T_MIT_. Therefore, to minimize the strain induced effect by lattice mismatch, to avoid the worsened quality of VO_2_, and to probe the effect of VO_2_ on graphene layer during the phase transition, the studies of graphene/VO_2_ heterostructure in this work were conducted by transferring graphene layers on top of high-quality VO_2_ layers fabricated on sapphire substrates instead. This work focus on probing the graphene/VO_2_ crystal structure and electrical properties during the phase transition of VO_2_ via temperature-dependent Raman spectroscopy and resistivity measurement, respectively. Slight increases in T_MIT_ of VO_2_ with graphene layer on top were observed. The G-peak splitting, which is a reversible process, at temperature near T_MIT_ of VO_2_ and higher was also observed and reported in this work for the first time, along with the unusual 2D-peak characteristics of graphene on VO_2_. Raman spectra of VO_2_ are also measured to probe the structural changes of the heterostructure. The results from the temperature-dependent of VO_2_ peak intensity are discussed and compared to the temperature-dependent resistivity measurement, while VO_2_ peak shift with the change in temperature results are included in the supplementary material. Our findings expand the knowledge about the mechanism of the graphene/VO_2_ heterostructure and may be useful for future applications, such as flexible thermochromic windows and the applications that requires a controllable structural graphene properties via temperature with the help of the VO_2_ phase transition.

## Results and discussion

**Figure 1:**
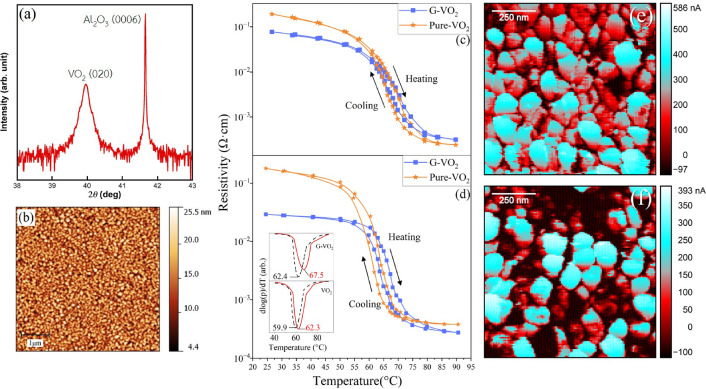
(**a**) Out-of-plane XRD scan and (**b**) 5 $\times$ 5 µm^2^ AFM surface scan of 100 nm VO_2_/*c*-Al_2_O_3_, respectively. The XRD and AFM scan results for 50 nm VO_2_/*c*-Al_2_O_3_ can be found in the supplementary material. The temperature-dependent resistance measurement result of CVD-graphene/VO_2_ samples with (**c**) 100 nm VO_2_ and (**d**) 50 nm VO_2_ (with $\frac{d(log(\rho ))}{dT}$ curve inset showing the T_MIT_ extracted from each cycle/sample area). C-AFM measurement results of CVD-graphene/VO_2_ (100 nm) sample on (**e**) graphene area and (**f**) pure-VO_2_ area.

The 50 and 100 nm VO_2_ thin films crystallinity and morphology were extracted using out-of-plane X-ray diffractometry (XRD) and atomic force microscopy (AFM), both results for the 100 nm VO_2_ film can be seen in Fig. 1a,b, respectively (the result for 50 nm VO_2_ can be found in supplementary material). The VO_2_ films possess single-crystalline structures. Two XRD peaks in Fig. 1a belong to VO_2_ (020) and *c*-Al_2_O_3_ (0006). The grain distribution of VO_2_ can be seen from the $\sim$ 5 $\times$ 5 $\upmu$m^2^ AFM image in Fig. 1b. The rms roughness of 100 nm VO_2_ is 2.6 nm. There are no pinholes or cracks on the surface for both 50 nm and 100 nm thin films. More XRD and AFM results for the 50 nm thin film can be seen in the supplementary materials. After confirmation of the crystallinity of VO_2_ films, graphene layers were transferred on top of the VO_2_ films, providing 6 samples in total (including the reference samples). A1 (CVD ref) and A2 (exf. ref) are graphene/*c*-Al_2_O_3_ reference samples from CVD growth and mechanical exfoliation, respectively. A3 (CVD/100) and A4 (CVD/50) are graphene(CVD)/VO_2_/*c*-Al_2_O_3_ with 100 nm and 50 nm VO_2_, respectively. A5 (exf./100) and A6 (exf./50) are graphene(exfoliated)/VO_2_/*c*-Al_2_O_3_ with 100 nm and 50 nm VO_2_, respectively. Note that A3–A6 samples have both pure-VO_2_ area and graphene/VO_2_ area available for Raman and resistivity measurements.

### Temperature-dependent resistivity measurement

The temperature-dependent resistivity on the samples A3 (CVD/100) and A4 (CVD/50) were measured in-plane and plotted in Fig. 1c,d, respectively. Both measurements include graphene/VO_2_ and pure VO_2_ area. There are several noticeable features in the results. Firstly, the resistivity dropped at the initial of the measurement (before the transition) for graphene/VO_2_ around $\sim$ 3 times compared to pure VO_2_, which may be attributed to the fact that graphene conductivity is much higher than the VO_2_ in the insulating phase^20^, hence the in-plane resistivity measurement is simply measuring the parallel two-resistor model, resulting in lower equivalent resistance. The conductive atomic force microscopy (C-AFM) current mapping results at room temperature in Fig. 1e (graphene area) and f (pure-VO_2_ area) from sample A3 (CVD/100) also confirm that graphene can improve the overall conductivity of the sample. It could also be the effect of electron injection into the VO_2_ layer due to the proximity of graphene, as reported by Zhou et al.^16^. Secondly, the phase transitions are less sharp on G-VO_2_ area compared to pure-VO_2_ area. Thirdly, the T_MIT_ is slightly higher for graphene/VO_2_ compared to pure VO_2_. The T_MIT_ from each sample was calculated by averaging the transition temperature in the heating and cooling cycles using the following equation:

1 $$\begin{aligned} T_{MIT}=\frac{T_{heating}+T_{cooling}}{2} \end{aligned}$$

where T$_\text {heating (cooling)}$ are the critical phase transition temperature calculated by finding the minimum value of $\frac{d(log(\rho ))}{dT}$ in heating (cooling) cycle. The T_MIT_ extracted from sample A3 (CVD/100) (Fig. 1c)for graphene/VO_2_ and pure VO_2_ area are 69.3 ^∘^C and 67.4 ^∘^C, respectively. The T_MIT_ extracted from sample A4 (CVD/50) (Fig. 1d) for graphene/VO_2_ and pure VO_2_ area are 64.9 ^∘^C and 61.1 ^∘^C, respectively. The $\frac{d(log(\rho ))}{dT}$ plot of sample A3 is shown in Fig. S3a and the plot of sample A4 is shown in the inset of Fig. 1d and S3b. In general, T_MIT_ from 50 nm VO_2_ sample is less than 100 nm VO_2_ sample because thinner VO_2_ film is subjected to higher compressive strain in c_R_ direction from VO_2_-*c*-Al_2_O_3_ lattice mismatch, which has been reported to affect the T_MIT_^21^. In contradiction to previous works done by Kim et al.^15^ and Zhou et al.^16^, the proximity of graphene results in higher T_MIT_ of VO_2_ in our work. According to the work from Kim et al., T_MIT_ can be reduced with the presence of graphene support layer underneath^15^. And the more graphene layers, the lower T_MIT_ observed in VO_2_^15^. It was proposed that the thermal expansion mismatch of the heterostructure may induce compressive strain in c_R_ (in-plane) direction. The thermal expansion coefficient ($\alpha$) of graphene is known to be negative around $-3.26 \times 10^{6}K^{-1}$^22^ and the $\alpha$ of VO_2_ are known to be positive. Therefore, the mismatch between $\alpha$ can induce the compressive strain in VO_2_. Another possible reason for lower T_MIT_ is the compressive strain on VO_2_ induced by lattice mismatch between graphene and VO_2_ in the growth process. With the reverse layer ordering presented in our work, the latter effect can be diminished. Another work from Zhou et al.^16^ proposed that the electron injection from graphene can occur between the graphene/VO_2_ interface, which destabilizes the insulator phase of VO_2_ and decreases T_MIT_. This phenomenon was observed in VO_2_/graphene/Ge heterostructure and described that the different in Fermi levels, which are Ge > graphene > VO_2_, introduce the Schottky barrier at each interface, resulting in electron flow from Ge to graphene and from graphene to VO_2_. Since the decrease in T_MIT_ cannot be observed in this work, it may indicate that the electron donor layer, such as Ge, is required for such an effect to occur. Also, it is noteworthy that the measurement technique for extracting the T_MIT_ may affect the T_MIT_ itself. Zhou also proposed that the measurement technique involving light radiation may introduce a higher carrier density, destabilize the insulator phase, and affect T_MIT_ even more. As for the increase in T_MIT_ observed in our work, due to the fact that the in-plane thermal conductivity of the graphene on Si substrate is about 500–1000 Wm^-1^K^-1^^23^ and VO_2_ is about 3–6 Wm^-1^K^-1^^24^, the graphene upper layer may act as a heat sink to the VO_2_ film, which can dissipate the heat out of the film. That resulted in a higher temperature required for the film to reach the actual T_MIT_ of VO_2_. As seen in Fig. 1c,d, the effect of graphene on the rise of T_MIT_ is more pronounced in the thinner VO_2_ film. The T_MIT_ of VO_2_ is increased by 1.9 ^∘^C for sample A3 (CVD/100) and 3.8 ^∘^C for sample A4 (CVD/50). This supports the hypothesis that the heat may be dissipated via the graphene layer, and the thinner VO_2_ layer, the higher heat dissipation efficiency, resulting in a larger decrease in T_MIT_.

**Figure 2:**
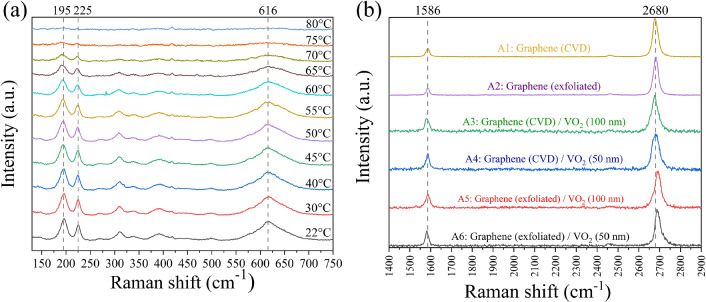
(**a**) Temperature-dependent Raman spectra of VO_2_ sample, the Raman intensity of VO_2_ decreases as temperature increases, and the VO_2_ peaks completely disappear as temperature reaches $\sim$70 ^∘^C (**b**) Raman spectra of all graphene samples on VO_2_/*c*-Al_2_O_3_ substrate, the reference samples are samples A1 (CVD ref) and A2 (exf. ref).

### Temperature-dependent Raman spectroscopy

The temperature-dependent Raman spectra of 100 nm VO_2_/*c*-Al_2_O_3_ film were measured as shown in Fig. 2a. At low temperature, the Raman spectra show the signature of VO_2_ monoclinic phase including 195, 225, 616 cm^-1^ vibrational modes. 195 and 225 cm^-1^ modes relate to V–V vibrational modes, while $\sim$ 616 cm^-1^ mode relates to V–O vibrational mode.^25^ As the temperature increases, all VO_2_ Raman peaks intensity starts decreasing at $\sim$ 55 ^∘^C and completely disappears at $\sim$75 ^∘^C, confirming the structural transition from monoclinic to rutile since the rutile phase is Raman inactive. VO_2_ samples were cut into $\sim$ 1 $\times$ 1 cm^2^ and graphene layers were transferred on top of *c*-Al_2_O_3_ and VO_2_/*c*-Al_2_O_3_ samples using two techniques as described in the methods section.

**Figure 3:**
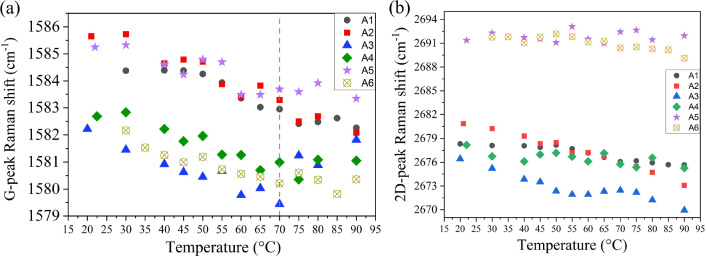
(**a**) Temperature-dependent G-Peak position of graphene on all samples (**b**) Temperature-dependent 2D-peak position of graphene on all samples.

**Figure 4:**
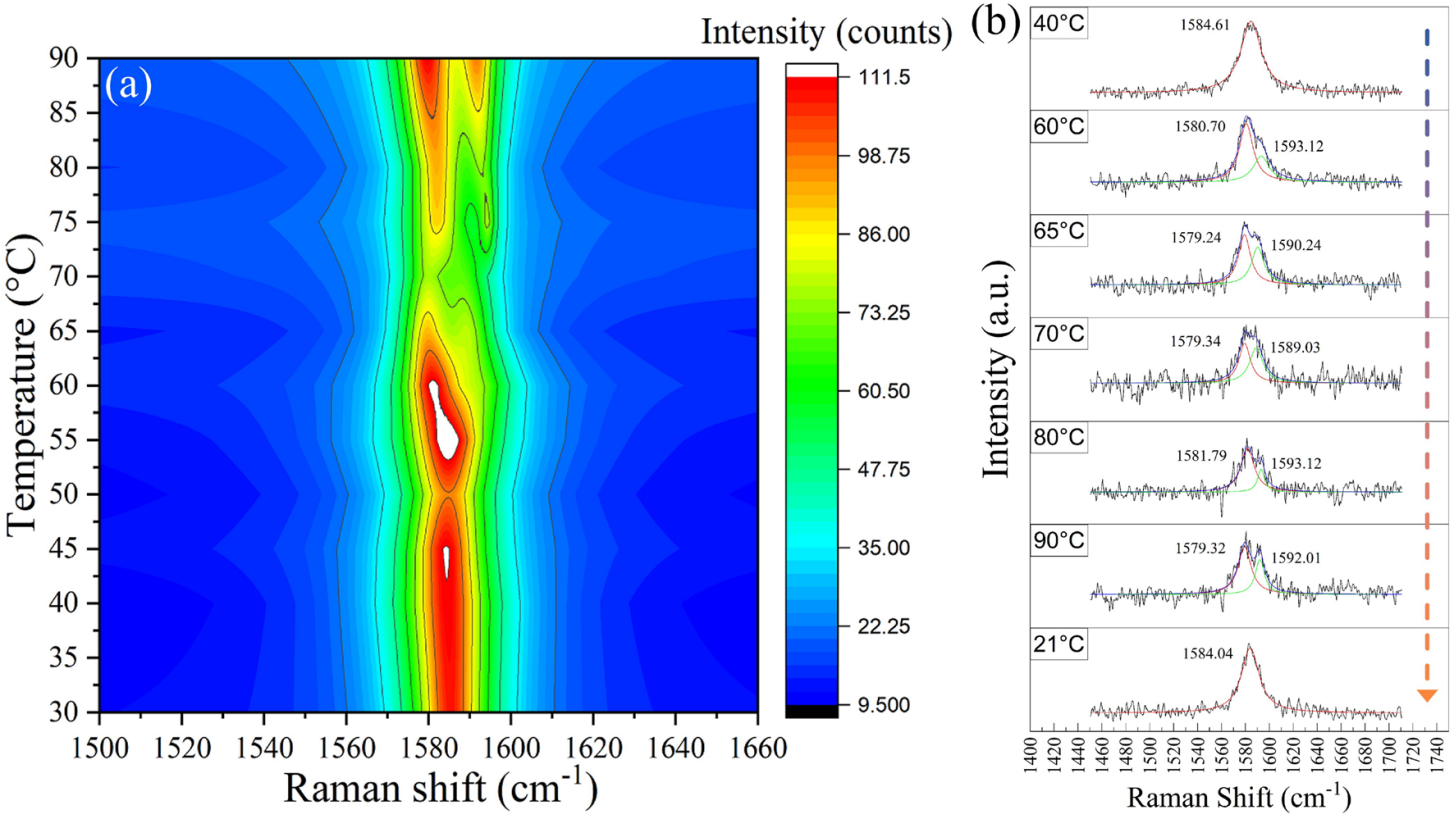
(**a**) Contour plot of graphene Raman shift from sample A5 (exf./100), the G-peak splitting starts showing at  60 ^∘^C (**b**) The evolution of G-peak splitting on sample A5 (exf./100) as temperature increases from 40 to 90 ^∘^C. As the sample was cooled down to 21 ^∘^C, the G-peak splitting disappeared, which can be considered as a reversible process.

Raman spectra of all samples (A1–A6) in graphene region are shown in Fig. 2b. The G and 2D peaks of each graphene are located at 1582–1595 cm^-1^ and 2676–2695 cm^-1^, respectively. The D-peaks at $\sim$ 1350 cm^-1^ of all samples are almost invisible, which indicates their good quality^26^. Figure 3a shows the relationship between G-peak position and temperature. The initial G-peak positions for each sample at room temperature (∼22 ^∘^C) are spatially dependent due to the local strain and defects on VO_2_ and graphene itself, which are unavoidable. The trends of G-peak shift, however, should be related to the interaction between graphene and VO_2_. Figure 3a shows that the red-shift trends of G-peak occurred as temperature increases, but only within the temperature range of 20–70 ^∘^C and diminished at higher temperature for sample A3–A6. The differences in G-peak slope from the linear fit of temperature-dependent Raman peaks data in the 20–70 ^∘^C and 20–90 ^∘^C region shown in Table 1 can confirm the diminished of red-shift trend, where sample A3–A6 show smaller slope of G-peak in the range of 20–90 ^∘^C, while the reference samples (A1–A2) have similar slopes for both temperature ranges. Generally, the G-peak position of graphene tends to red-shift linearly as temperature increases as proposed in the theoretical work by Bonini et al.^27^, and also observed in several experimental works^28–30^. The red-shifts of the G-peak due to temperature can be expressed as contributions from several effects^30^:

2 $$\begin{aligned} \Delta \omega _G(T) =\Delta \omega _G^E(T) + \Delta \omega _G^A(T) + \Delta \omega _G^S(T) \end{aligned}$$

where ${\Delta \omega _G(T)}$ is the frequency shift of the G-peak, $\Delta \omega _G^E(T)$ is the shift from graphene thermal expansion, which is normally positive (blue-shift) due to the negative thermal expansion nature of graphene, $\Delta \omega _G^E(T)$ is the shift due to the self-energy shift from the 4-phonon interaction, which is the intrinsic effect in graphene^27^ and $\Delta \omega _G^S(T)$ is the shift due to the thermal expansion coefficient mismatch between graphene and its substrate. However, the observed phenomenon in this work is different. In addition to the diminished red-shift trend of G-peak, the splitting of G-peak was also observed as the temperature reached $\sim$ 60 ^∘^C for both CVD graphene and exfoliated graphene on VO_2_ (100 nm) samples (A3-CVD/100 and A5-exf./100). The contour of Raman spectra of G-peak at various temperatures for sample A5 (exf./100) can be seen in Fig. 4a. The evolution of G-peak with temperature can be seen in Fig 4b, as it was heated up (40–90 ^∘^C) then cooled down. The splitting occurred from G-peak at $\sim$ 1583 cm^-1^ into G^-^ around 1580 cm^-1^ and G^+^ around 1593 cm^-1^. The splitting disappeared after the sample was cooled down, indicating that this is a reversible process that originated from the presence of VO_2_. As for the CVD graphene sample, the G-peak splitting was also observed with some different details, as shown in Fig. S5 in the supplementary file. One possible explanation for this phenomenon is the asymmetric tensile strain applied to graphene induced by the phase transition of VO_2_ and its thermal expansion mismatch. The $\alpha$ of VO_2_ for in-plane direction were calculated by Théry et al.^25^ which are $\alpha _a^M=12.1\times 10^{-6}K^{-1}, \alpha _c^M=2.57\times 10^{-6}K^{-1}, \alpha _b^R=5.83\times 10^{-6}K^{-1}$ and $\alpha _c^R=29.7\times 10^{-6}K^{-1}$. Where the superscripts M and R indicate the monoclinic and rutile phases of VO_2_, and subscripts a, b, and c indicate the direction of the crystal. In terms of direction, $\alpha _a^M$ is equivalent to $\alpha _c^R$ and $\alpha _c^M$ is equivalent to $\alpha _b^R$. According to the theory proposed by Klimov et al.^31^, as the temperature increases, each grain of VO_2_ starts to change its phase from monoclinic to rutile. The phase transition for each grain may occur at temperature before or after reaching T_MIT_ depending on their grain size^31^. The C-AFM measurement results from sample A3 (CVD/100) (see Fig. 1e,f) show that the grain boundaries are varied for both graphene/VO_2_ and pure VO_2_ area. Combining with the fact that the $\alpha$ in each in-plane direction of VO_2_ are different, which $\alpha _c^R$ is significantly higher than the $\alpha _b^R$. Therefore, it is most likely that the in-plane tensile strain from VO_2_ at higher temperature tends to be asymmetric, resulting in the blue-shift of G-phonon mode in the direction with lesser tensile strain and red-shift in the higher tensile strain direction. The results presented are in good agreement with the work from Mohiuddin et al.^32^ which reports about the G-peak splitting due to uniaxial strain applied to the graphene on a flexible substrate.

Aside from the G-peak shifts, the 2D-peak measurement also yields interesting results (see Fig. 3b) The 2D-peak positions of samples A1–A4 are located in the range of 2669–2680 cm^-1^ while A5 (exf./100) and A6 (exf./50) are located between 2689 and 2693 cm^-1^ (blue-shifted by $\sim$ 13 cm^-1^ compared to ref. sample). Normally, the 2D-peak of graphene exhibits a red-shift trend, with an even lesser slope compared to G-peak red shift trend under increasing temperature and uniaxial-strain.^30,32^. However, in contrast to our G-peak result, the temperature-dependent 2D-peak trends observed from all samples except A3 (CVD/100) are almost constant with small fluctuations instead of red-shift as observed from the reference samples A1 (CVD ref) and A2 (exf. ref) as seen in Fig. 3b and Table 1. There should be more interaction between graphene and VO_2_ that modifies the electronic band structure of graphene, resulting in some modification of the double resonance Raman scattering process across the K-point of electronic dispersion, which is the origin of the 2D-peak^29,33,34^. Further experimental and theoretical studies are required to provide further analysis.

**Table 1:** Slope of temperature-dependent Raman measurement on G and 2D peaks from all samples in cm^-1^ ^∘^C^-1^.

Sample	G-peak (20–70 ^∘^C)	G-peak (20–90 ^∘^C)	2D-peak (20–70 ^∘^C)	2D-peak (20–90 ^∘^C)
A1: CVD graphene	−0.04270	−0.04332	−0.04052	−0.04583
A2: Exf. graphene	−0.05262	−0.05635	−0.10135	−0.10989
A3: CVD graphene/100 nm VO_2_	−0.05036	−0.01061	−0.08842	−0.07852
A4: CVD graphene/50 nm VO_2_	−0.04483	−0.03345	−0.02709	−0.02914
A5: Exf. graphene/100 nm VO_2_	−0.03883	−0.03023	0.00455	0.00482
A6: Exf. graphene/50 nm VO_2_	−0.04223	−0.02903	−0.02295	−0.03875

**Figure 5:**
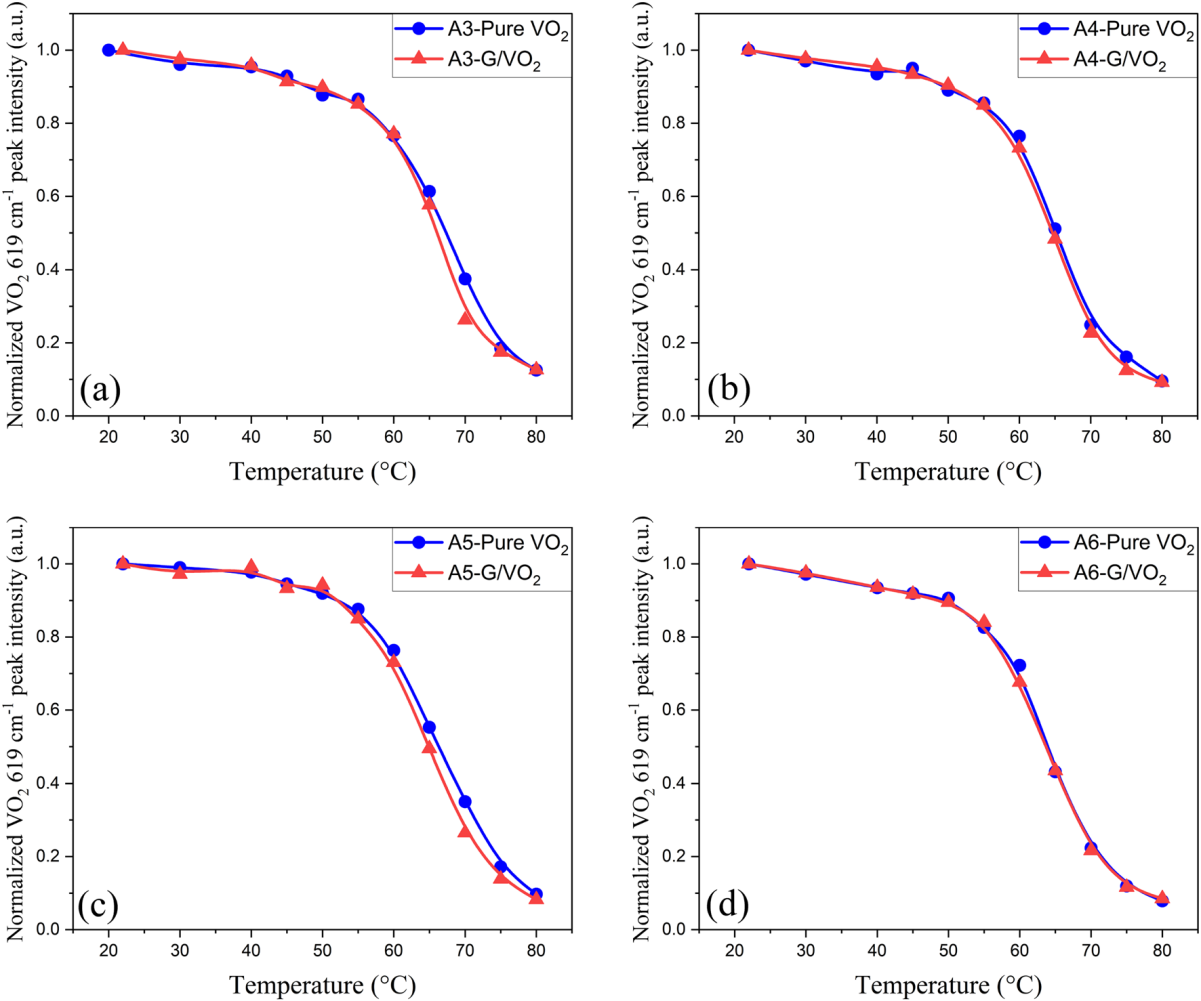
Temperature-dependent normalized VO_2_ 616 cm^-1^ Raman intensity from sample (**a**) A3 (CVD/100) (**b**) A4 (CVD/50) (**c**) A5(exf./100) and (**d**) A6 (exf./50).

Temperature-dependent Raman spectra of VO_2_ vibrational modes were also measured and compared on sample A3–A6 on both graphene/VO_2_ and pure VO_2_ areas. Three main vibrational modes Raman shifts of VO_2_, $\sim$ 195, $\sim$ 225 and $\sim$ 616 cm^-1^ mode, were plotted against temperature as shown in Fig. S4 in supplementary material. The trend lines between each sample are different from each other, but the trend lines observed from the same sample are almost identical except at temperatures higher than 65 ^∘^C where their Raman spectra might be too noisy (VO_2_ intensity drop sharply at 65–70 ^∘^C). The similarity between the results from graphene/VO_2_ area and pure-VO_2_ area observed from sample A3–A6 as shown in Fig. S3 indicates that graphene layer might have no effect on VO_2_ structural properties. Figure 5 shows the temperature-dependent normalized VO_2_ $\sim$ 616 cm^-1^ Raman intensity for sample A3-A6 in the heating cycle. In general, the structural phase transition curve remains almost unchanged or slightly shifts to the left, indicating that the graphene layer provides almost no effect on T_SPT_. However, there might be a slight decrease in T_SPT_ for the thicker VO_2_ (samples A3 and A5 as seen in Fig. 5a,c, respectively). The reason is that the 100 nm VO_2_ is more relaxed from the substrate clamping effect compared to the 50 nm, hence the presence of graphene on top has more effect^10^. Therefore, the increase in T_MIT_ as discussed earlier may not originate from structural changes in VO_2_. The increase in T_MIT_ is likely to originate from other factors such as the different thermal conductivity between two layers and the phase transition mechanism of VO_2_ as discussed in the previous section.

## Conclusion

In order to understand the interaction between graphene and VO_2_, temperature-dependent resistivity and Raman measurements were performed on graphene/VO_2_/*c*-Al_2_O_3_. The samples were prepared by fabricating VO_2_ using the RBTIBD technique, followed by wet transfer (for CVD graphene) or mechanical exfoliate (for exfoliated graphene) the graphene layer onto VO_2_ layer. In contrast to other previous work^15,16,35^, our samples layer ordering were swapped to graphene layer on top of VO_2_ in order to study the heterostructure without worsening graphene and VO_2_ layer along with minimizing the strain effect induced by lattice mismatch between graphene and VO_2_. From the resistivity measurement, the less sharp phase transition characteristics and higher T_MIT_ of VO_2_ on graphene/VO_2_ area compared to pure VO_2_ area were observed. The T_MIT_ in graphene/VO_2_ samples slightly increase by $\sim$ 1.9 ^∘^C and $\sim$ 3.8 ^∘^C compared to pure VO_2_ for sample with 100 nm and 50 nm VO_2_, respectively. This might originate from a large difference between the thermal conductivity of graphene and VO_2_, resulting in the graphene layer act as a heat sink, thus the VO_2_ layer requires slightly higher temperature than T_MIT_ to change its phase. The effects of VO_2_ phase transition on the graphene layer were observed, as seen in the G-peak splitting from temperature-dependent Raman measurements for the first time. The G-peak splitting is attributed to the asymmetric strain applied to graphene due to the thermal expansion mismatch between graphene and VO_2_ during the VO_2_ phase transition. The asymmetric strain may originate from the fact that each grain of VO_2_ starts to change its phase at different temperatures. Thus, the strain from the thermal expansion of VO_2_ can affect graphene in random direction and cause asymmetric changes of graphene structure. The 2D-peak Raman measurement results also show a large blue-shift of about 13 cm^-1^ for exfoliated graphene on VO_2_ and the absence of red-shift trends in all graphene/VO_2_ samples except for sample A3 (CVD/100). There should be more electronic band interaction between graphene and VO_2_ that requires further experiment to clarify. These findings may provide a better understanding of the heterostructure and introduce some possibilities for the application of graphene/VO_2_ which utilizes the controllable structural properties of graphene with the help of VO_2_ phase transition.

## Methods

Two VO_2_ thin films were deposited on top of *c*-Al_2_O_3_ substrates using the RBTIBD method. The deposition process and conditions can be found elsewhere.^36^ The estimated thickness of the VO_2_ layers measured by X-ray Reflectivity (XRR) technique are $\sim$ 50 nm and $\sim$ 100 nm. The surface morphology of the films and crystal structure were analyzed via AFM, XRD and Raman spectroscopy techniques. The graphene layers were transferred onto VO_2_/*c*-Al_2_O_3_ and *c*-Al_2_O_3_ reference substrates using two methods: mechanical exfoliation via the scotch-tape method and the CVD graphene wet-transfer method. The first technique was performed with a natural graphite flake by spreading a few layers of graphene across a scotch-tape, then press them on the VO_2_ layer. The exfoliation steps are slightly modified from Huang et al.^37^. The latter method was performed with CVD graphene bought from Graphenea by submerging graphene and the PMMA layer into DI water to separate the graphene/sacrificial layer from the PMMA. Then, the VO_2_/*c*-Al_2_O_3_ samples were dipped and fished up the graphene layer, followed by heating treatment and acetone cleaning. More detail on both transfer methods can be found in the supplementary material. The characteristics related to the phase transition of VO_2_ were analyzed using the temperature-dependent Raman spectroscopy technique with a 532 nm excitation laser wavelength. The excitation laser power in this work is less than $\sim$ 0.5 mW in order to avoid heat damage on graphene and VO_2_. The grating for Raman measurement is 1800 lines/mm. Raman peaks captured from the temperature-dependent Raman results were fitted with the Lorentzian function. As for the temperature-dependent resistivity measurement, the 100 nm-thick Au electrodes were fabricated on VO_2_ samples with CVD-graphene using UV-lithography and metal evaporation techniques. The measurements were conducted to probe the electrical properties of the Graphene(CVD)/VO_2_ heterostructure. The temperatures were measured on mini-hot plate via thermocouple with temperature error of ± 1 ^∘^C.

## Supplementary Information

Supplementary Information.

## Data Availability

The datasets used and/or analysed during the current study available from the corresponding author on reasonable request.
