# Supplementary material for: Metal-insulator transition effect on Graphene/VO2 heterostructure via temperature-dependent Raman spectroscopy and resistivity measurement
Source: Sci Rep. 2024 Feb 24;14:4545. doi: 10.1038/s41598-024-54844-w (PMC11636808; doi:10.1038/s41598-024-54844-w)
Supplement: Supplementary file 1 — Supplementary Information. [file 41598_2024_54844_MOESM1_ESM.pdf]

Supplementary material: Metal-insulator transition effect on Graphene/VO_2 heterostructure via temperature-dependent Raman spectroscopy and resistivity measurement

Kittitat Lerttraikul[1], Wirunchana Rattanasakuldilok[2], Teerachote Pakornchote[1], Thiti Bovornratanaraks[1], Illias Klanurak[1], Thiti Taychatanapat[1], Ladda Srathongsian[3], Chaowaphat Seriwatanachai[3], Pongsakorn Kanjanaboos[3], Sojiphong Chatraphorn[1], and Salinporn Kittiwatanakul[1,*]

[1]Department of Physics, Faculty of Science, Chulalongkorn University, Bangkok 10330, Thailand
[2]Department of Physics, Accelerator laboratory, University of Jyvaskyla, P.O. Box 35(YFL), 40014 University of Jyvaskyla, Finland
[3]School of Materials Science and Innovation, Faculty of Science, Mahidol University, Nakhon Pathom 73170, Thailand
[*]salinporn.k@chula.ac.th

Graphene transfer method

In this work, two methods were performed in order to transfer the graphene layer onto VO_2 thin films. The first method, which is used for samples A1 (CVD ref), A3 (CVD/100) and A4 (CVD/50) samples, is CVD wet-transfer, in which CVD graphene layers were ordered from Graphenea. Normally, the graphene layers are attached to the sacrificial layer on top and placed on thin paper. The detailed steps for transferring graphene are:

1. Clean the ~1x1 cm^2 VO_2/c-Al_2O_3 samples using a sonicator in methanol for 25 minutes and isopropanol (IPA) for 5 minutes, dry up the samples using a nitrogen gun, then bake on a hot plate at 100°C for 1 minute
2. Clean the VO_2/c-Al_2O_3 samples again with plasma etching technique under oxygen atmosphere at pressure of 130 mtorr for 5 minutes
3. Cut the sacrificial layer/graphene on paper into small pieces around ~0.5×0.5 cm^2
4. Float the sacrificial/graphene layer with paper in deionized water, the PMMA/graphene layer will be detached from the paper
5. Dip the VO_2/c-Al_2O_3 sample into the deionized water surface at 45° angle, slowly swing up the sample to attach the sacrificial/graphene layer on top of VO_2 layer, and leave it at room temperature for 1 hour (see fig. S1a.)
6. Bake the sacrificial/graphene/VO_2 sample on a hotplate at 100°C for 1 hour
7. Place the sample inside a vacuum-sealed container to allow the graphene/PMMA to adhere tightly to VO_2 sample for 1 day
8. Remove the sacrificial layer by dipping the sample into a hot acetone bath at 50°C for 1 hour, then IPA at room temperature for 1 hour, blow-drying it with a nitrogen gun and bake it on a hotplate at 100°C for 1 minute.

After this process, one could observe wrinkles on the graphene layer using an optical microscope (see fig. S1b.). However, most of the graphene area might not have wrinkles, which can only be confirmed by the Raman spectroscopy technique. It can also be used to confirm the integrity and quality of the graphene layer. Another method, which is used for samplse A2 (Exf, ref), A5 (Exf./100) and A6 (Exf./50), is the scotch-tape dry exfoliation method. The steps to reproduce the samples used in this work are:

1. Prepare scotch tape with low residue i.e., blue tape, cut it in a horizontal direction

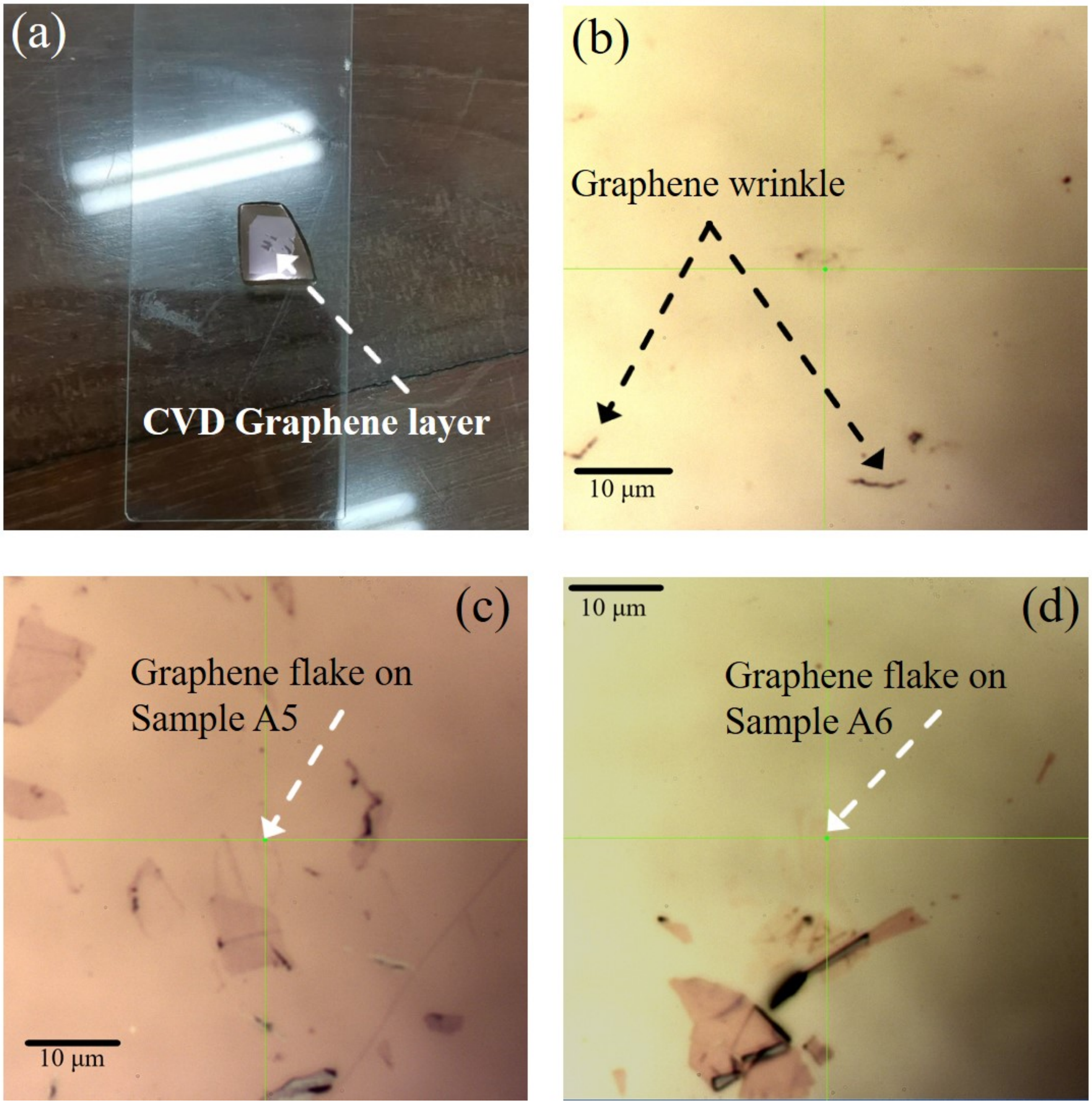

Figure S1. (a) CVD graphene layer on the substrate, the graphene area can be easily observed due to the presence of sacrificial layer (b) CVD graphene area which cover the whole image, some area may contain graphene wrinkle as shown in the figure (c) Graphene flake from sample A5 (Exf./100) (d) Graphene flake from sample A6 (Exf./50). Both A5 and A6 graphene flake often surrounded by many multilayer-graphene or graphite flake, the number layer of each flake must be confirmed using Raman spectroscopy technique

2. Gently press the natural graphite flake (shiny side) onto the leftmost side of the scotch tape, pull scotch tape against table rim to make the graphite flake lift a little bit, remove it with a tweezer, gently press it again nearby the previous area, keep doing it until small flake of graphite has around 1x1 inch^2 on scotch tape
3. Sandwich the leftmost side of the scotch tape onto the rightmost side and peel scotch tape off, keep doing it on the area with no graphite flake until the tape is filled with graphite flake, this tape can be called "mother tape" which can be used to create 2-3 "daughter tape" for later exfoliation
4. Make daughter tape by preparing another scotch tape with the same size, carefully sandwiching it on mother tape from the leftmost to the rightmost side, avoid letting air bubble in between the two tape
5. Clean the $\sim$1×1 cm^2 $VO_2/c\text{-}Al_2O_3$ samples using a sonicator in methanol for 25 minutes and isopropanol (IPA) for 5 minutes, dry up the samples using a nitrogen gun and bake on a hot plate at 100°C for 1 minute
6. Clean the $VO_2/c\text{-}Al_2O_3$ samples again with plasma etching technique under oxygen atmosphere at pressure of 130 mtorr for 5 minutes
7. Carefully press the daughter tape onto $VO_2/c\text{-}Al_2O_3$ from left side to right side, avoiding bubbles between tape and substrate
8. Lift the tape with the sample and put it on top of 100°C hot plate and bake it for 1 minute, then carefully lift the tape and sample and cooled them down to room temperature for 5 minutes (keep both the scotch tape and sample attached together)
9. Pull the tape at both ends and gently press the tape area with the sample under it, gently remove the sample from the tape

Many graphene and graphite small flake can be seen on the $VO_2/c\text{-}Al_2O_3$ substrate after the process (Fig. S1c. and S1d.). One could search for the flake with high opacity (almost invisible) for graphene area, then confirm with the Raman spectroscopy technique to determine the number of layers of graphene flake.

VO_2 thin film characterization

Out-of-plane XRD and 5×5 μm^2 AFM scans from 100 nm and 50 nm VO_2 samples were reported in Fig. S2. The observed XRD peaks for both samples originated from the (020) plane of VO_2 orientation due to the same fabrication process (RBTIBD as mentioned in the main text) except for the deposition time. However, the surface roughness and the grain size between 100 nm VO_2 and 50 nm VO_2 are different; 100 nm sample has higher surface roughness and grain size. These characteristics originated from the relaxation of substrate clamping effect for thicker film.

Temperature-dependent VO_2 Raman peaks on graphene-VO_2 heterostructure

Temperature-dependent VO_2 Raman spectra were measured on graphene/VO_2 samples (A3-A6) on both pure VO_2 and graphene/VO_2 for every sample, as shown in Fig. S4a. for $\sim$195 cm^{-1}, S4b. for $\sim$225 cm^{-1} and S4c. for $\sim$616 cm^{-1} VO_2 peak. The $\sim$195 and $\sim$225 cm^{-1} peaks are attributed to V-V bonding; they are slightly different due to the zigzag V-V bonding observed in the monoclinic phase. The $\sim$616 cm^{-1} peak is attributed to V-O bonding. All three peaks are red-shifted as temperature increases due to the thermal expansion causing the relaxation of V-V and V-O bonding, resulting in lower peaks frequency. However, the presence of graphene layers does not affect any significant change in VO_2 peak temperature-dependent results, maybe due to the fact that graphene is only one layer while the Raman spectroscopy technique measures the average change from many layers of VO_2 (depending on the penetration depth).

Peak-splitting observed on Graphene(CVD)/VO_2 sample (sample A3-CVD/100)

Fig. S5 shows the evolution of G-peak for sample A3 (CVD/100). As the temperature increases, the G-peak characteristic starts to show asymmetric features. The left-side bending of the G-peak at 70°C and the G-peak splitting at 75°C and 80°C were observed. The splitting and bending are diminished as the temperature decreases to 23°C, which indicates that this is a reversible process. The G-peak splitting observed on sample A3 does not occur continuously, as seen in sample A5 (exf./100) (Fig. 4b.). The difference in the peak evolution between A3 and A5 may depend on the different origins of CVD and exfoliated graphene. CVD graphene layers were fabricated with large area coverage, while exfoliated graphene layers were transferred as small flakes. Thus, the grain size of CVD graphene tends to be larger than that of exfoliated graphene, resulting in a more uniform strain distribution during VO_2 phase transition. This is in agreement with our result from Fig. S5 that the G-peak splitting can only be observed at 75°C and 80°C, the temperature at which VO_2 tends to expand asymmetrically the most.

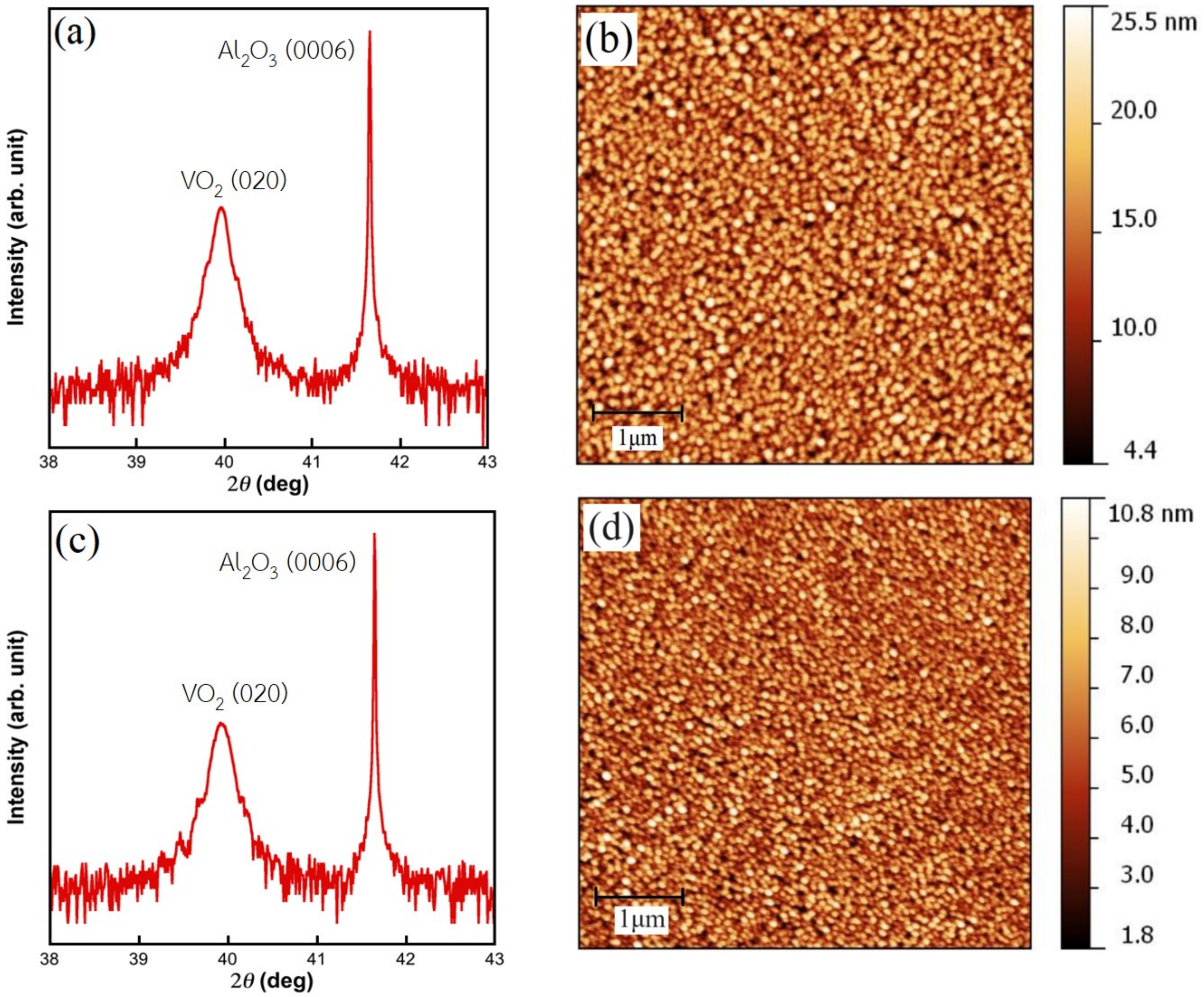

Figure S2. Out-of-plane XRD scans and 5×5 μm^2 AFM surface scans for (a,b) VO_2 (100 nm)/*c*-Al_2O_3 and (c,d) VO_2 (50 nm)/*c*-Al_2O_3. The average surface roughness are 1.5 nm and 2.6 nm, respectively.

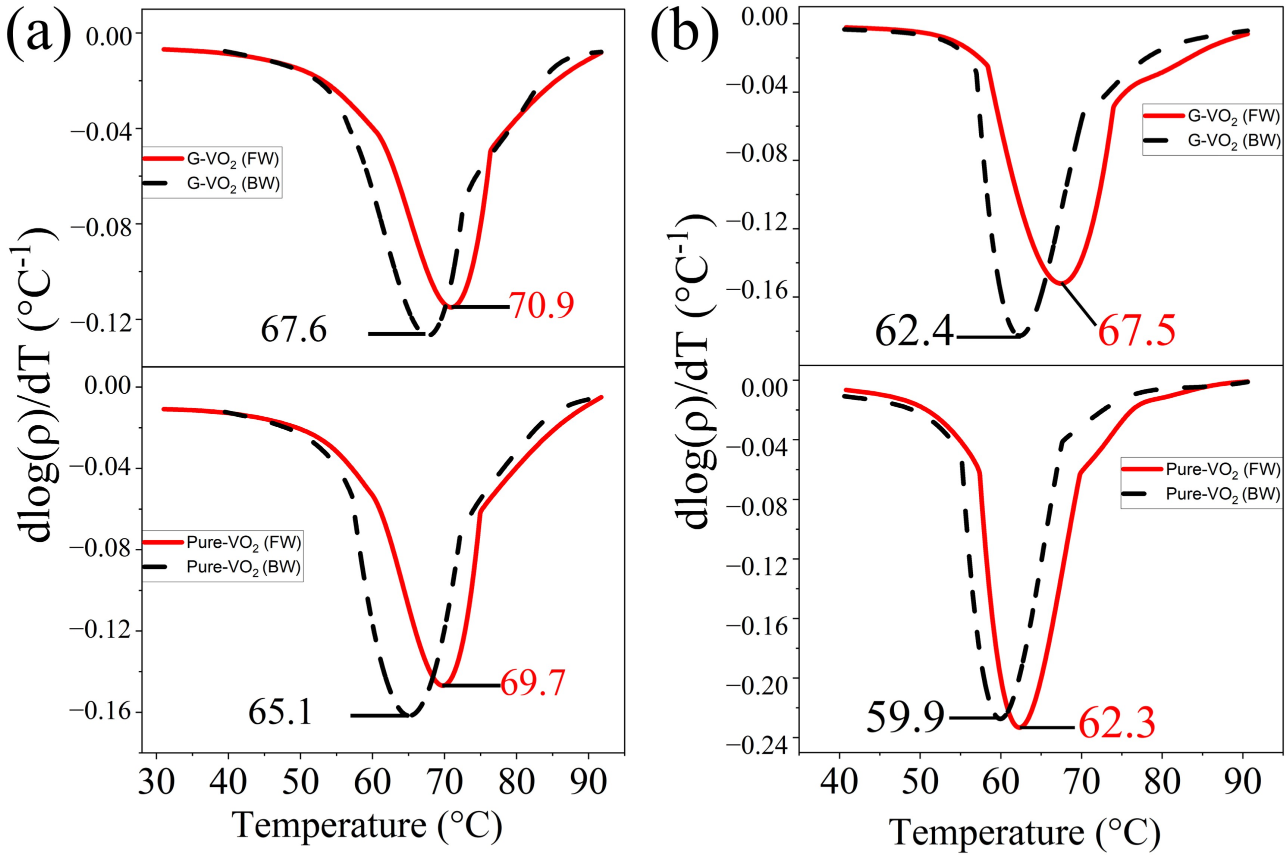

Figure S3. $\frac{d(log(\rho))}{dT}$ curve of the sample (a) A3 (CVD/100) and (b) A4 (CVD/50), showing the extracted T_{MIT} of each heating and cooling cycle. The T_{MIT} results were calculated by fitting the logarithm of resistivity data using a fourth-degree polynomial within the temperature range from 55-75 °C, followed by finding the minimum value from the differentiated equation.

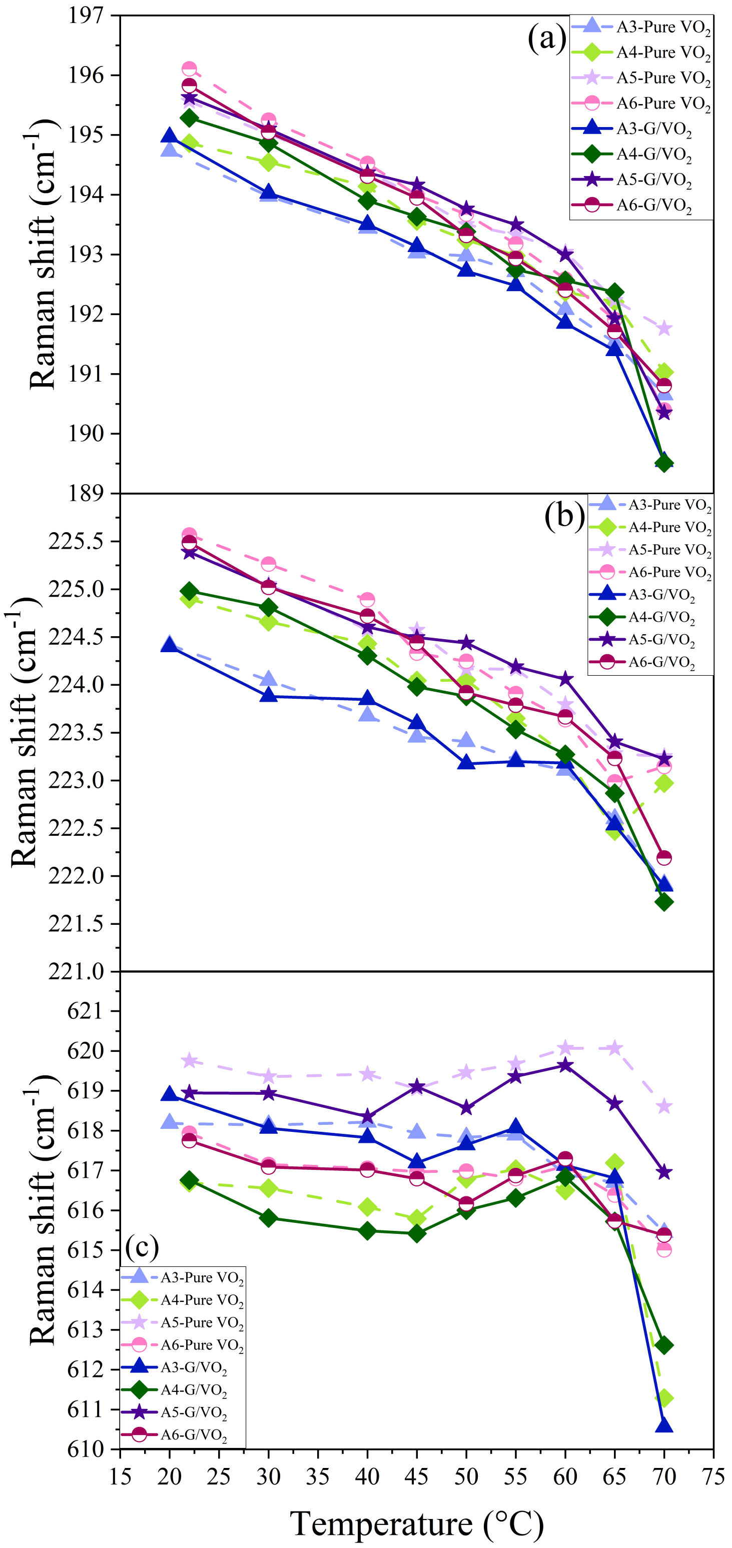

Figure S4. Temperature-dependent Raman measurement extracted from three main VO_2 peak: (a) $\sim$195 cm^{-1} peak, (b) $\sim$225 cm^{-1} peak and (c) $\sim$616 cm^{-1} peak.

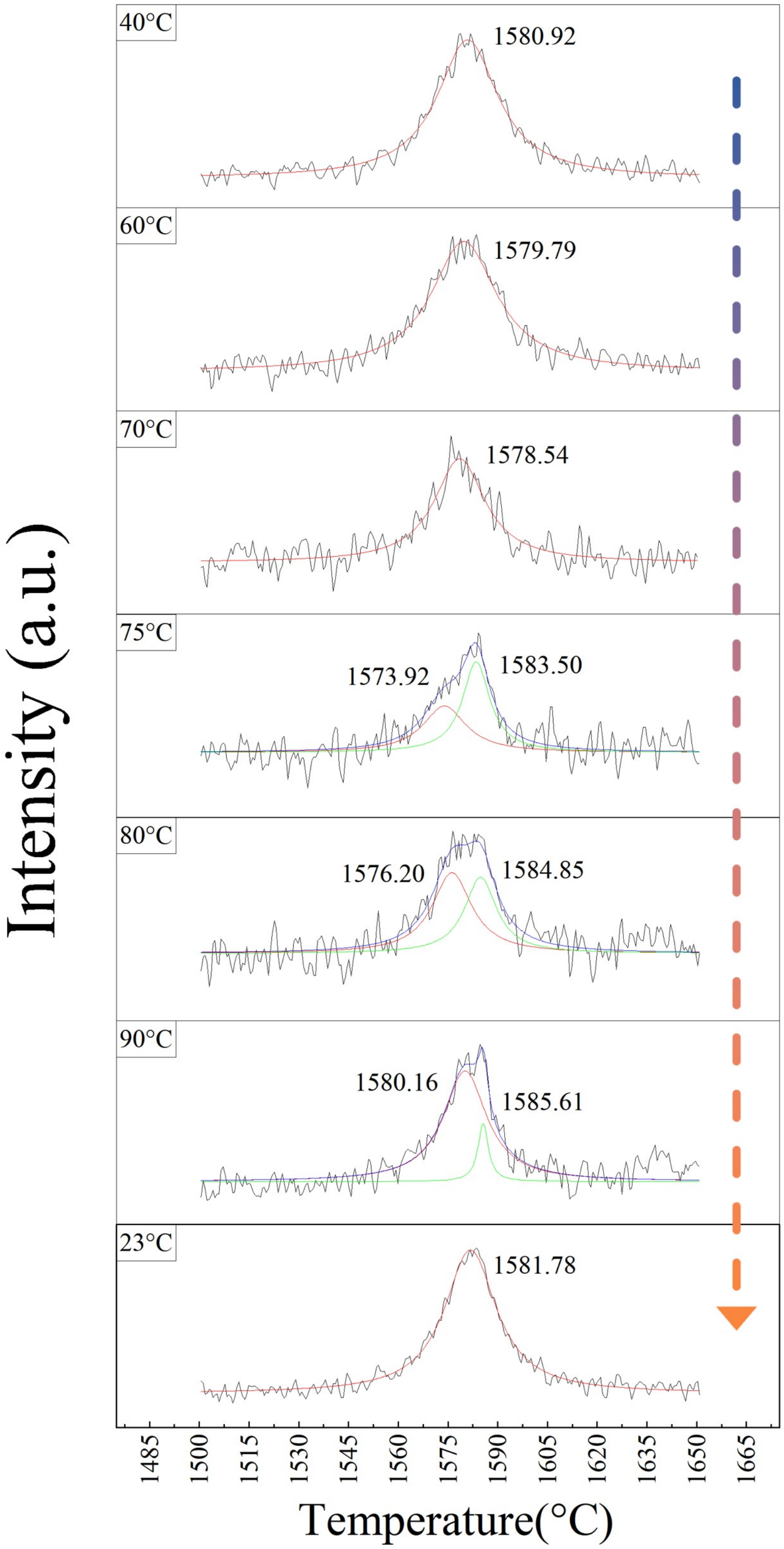

Figure S5. The evolution of G-peak splitting observed from sample A3(CVD/100), the G-peak begin to show asymmetric feature at 70°C (G-peak bending left) and the peak splitting occurred at 75°C and 80°C.
